# Characteristics of COVID and post COVID polyneuropathies in adults and pediatrics: an Egyptian sample

**DOI:** 10.1186/s41983-021-00435-9

**Published:** 2021-12-20

**Authors:** Haidy Elshebawy, Mohamed Y. Ezzeldin, Eman Hassan Elzamarany

**Affiliations:** 1 Neurology Department, Faculty of Medicine, Cairo University, Cairo, Egypt; 2 Present Address: Neuropsychiatry Department, Faculty of Medicine, South Valley University, Qena, Egypt; 3 Neurophysiology Department, Faculty of Medicine, Cairo University, Cairo, Egypt

**Keywords:** Post COVID, Polyneuropathies, Adults, Pediatrics

## Abstract

**Background:**

The aim of this study is to describe the different forms of polyneuropathy associated with coronavirus disease 2019 **(**COVID-19) as a secondary neurological complication for **(**COVID-19) and the outcome from different therapeutic regimens in adults and pediatrics in first and second waves of the pandemic.

**Case presentation:**

This study was conducted on 42 patients, they were divided into two groups, group (A) and group (B) in first and second waves respectively. Twenty-five patients presented by ascending weakness preceded by fever, dry cough and respiratory distress, electromyography (EMG) and nerve conduction (NC) studies done and confirmed the clinical diagnosis of demyelinating polyneuropathy. Eight patients presented by acute flaccid quadriparesis, more severe in upper limbs preceded by fever and diarrhea diagnosed as acute axonal polyneuropathy. Five patients presented by severe fatigue and progressive weakness of both lower and upper limbs, they developed fever and cough 10 days after the neurological symptoms. EMG and NC done and confirmed clinical diagnosis of polyneuropathy of demyelinating with secondary axonal picture. Four patients presented 30 to 40 days after their recovery form corona virus infection with gradual progressive weakness of both upper and lower limbs over 2 to 3 months duration, mainly the proximal muscles of lower limbs were affected with areflexia. EMG and NC done and confirmed the diagnosis of chronic inflammatory demyelinating polyneuropathy **(**CIDP).

**Conclusion:**

We should gain a better understanding of the underlying pathophysiology and therapeutic options of polyneuropathies related to COVID-19, which will have an impact on the treatment of the COVID related respiratory failure presenting with neuropathy.

## Introduction

The novel coronavirus disease 2019 (COVID-19) pandemic is a world-shattering infection that affects all geographical areas. With the rapid increase in the numbers of cases of COVID-2 pandemic with its different waves, clinical presentation and complications are rising across the countries and the whole world since December 2019 [1].

Corona virus like many respiratory viruses exhibit respiratory as well as extra pulmonary symptoms, and known that it may include the nervous system and causing a variety of neurological manifestations through direct neural invasion or indirect virus-induced host immune responses [2].

COVID-19 associated neuromuscular disorders are the second most commonly encountered neurological complications, especially Gullian barre syndrome (GBS) [3]. Diagnosis and management of these complications will have an impact on the treatment of the severe acute respiratory syndrome coronavirus 2 (SARSCoV-2) related respiratory failure presenting with neuropathy [4]. The aim of this study is to describe COVID-19 patients who presented or complicated with different forms of polyneuropathy in adults and pediatrics in first and second waves of the pandemic and their outcome with different therapeutic regimens.

## Methods

Our study is a cross sectional descriptive study conducted on 42 patients presented to us in the Neurology outpatient clinics from multiple health care centers in Egypt by multiple clinical forms of polyneuropathy in period from March 2020 to end of February 2021. Patients recruited from the outpatient clinics were divided into two groups. Group (A) involved 23 patients recruited in the first wave of corona virus in the period from March 2020 till the end of September 2020 and group (B) involved 19 patients recruited in the 2nd wave in the period from October 2020 till February 2021.We included in our study patients who presented with new onset polyneuropathy in any age group following or complicating COVID 19 infection. Patients who have a previous history of polyneuropathy or have a chronic illness which may be associated with polyneuropathy eg. (DM, autoimmune disorders, HCV) were excluded. An informed written consent was taken from each patient. All data obtained from every patient were confidential and were not used outside the study. Our study was approved by ethical committee of the Department of Neurology, Faculty of Medicine (Cairo University).

All patients in our study were conducted for history taking, neurological examination, EMG and NC studies were carried out for all patients to confirm clinical diagnosis of polyneuropathy.

SARS-CoV-2 testing from nasal swab, computed tomography (Ct) chest and other laboratory investigations (CBC, ESR, CRP, ferritin and D-dimer); were done for all patients.

EMG and nerve conduction studies were done using Nihon Kohden Neuropack M1 MEP-9200 EMG machine, Japan. Posterior tibial, common peroneal, superficial peroneal, motor and sensory ulnar nerves were examined for NCS, bilaterally. Both Facial nerves were added for NC protocol of study if there were clinical facial signs. EMG examination was carried out for both tibialis anterior, vastus medialis, deltoid, biceps brachii and first dorsal interosseous muscles.

### Clinical presentations

Twenty-five patients presented by ascending weakness affecting both lower and upper limbs with severe hypotonia and areflexia after manifestations of fever, dry cough and headache by (5–10) days. Three of them developed respiratory distress. EMG and NC done for all of them fulfilling the criteria of acute demyelinating polyneuropathy, at least three of the following were found: prolonged DLs (two or more nerves, not at entrapment sites) DL > 115% of ULN (for normal CMAP amplitudes), DL > 125% of ULN (for CMAP amplitudes < LLN), CV slowing (two or more nerves, not across entrapment sites) CV < 90% of LLN (for CMAP amplitudes > 50% LLN) CV < 80% of LLN (for CMAP amplitudes < 50% LLN), Prolonged F responses (one or more nerves) > 125% of ULN; or absent F responses, in addition to conduction block and/ or temporal dispersion may be found. EMG examination showed MUP’s of reduced recruitment and no at rest activities. PCR was positive for COVID in nasopharyneal swap in all of them. All of them received eight sessions of plasma exchange, twenty of them improved with good outcome and discharged on physiotherapy and five partially improved and need intravenous immunoglobulins (IVIG) for 5 days with better outcome and discharged.

Eight patients presented by acute flaccid quadriparesis, more severe in upper limbs mainly distal with areflexia, preceded by fever and diarrhea 1 week before the neurological manifestations, MRI cervical spine was free for all of them, EMG and NC showed picture of acute axonal polyneuropathy in form of reduced amplitudes of CMAPs (compound motor action potentials) and SNAPs (sensory nerve action potentials) and slightly reduced conduction velocities not reaching the demyelinating range, MUP’s of reduced recruitment and resting activities in the form of positive sharp waves and fibrillation potentials may be found in EMG. Also, PCR from nasal swap and other laboratory data confirm COVID diagnosis. Five of them received one cycle of IVIG for 5 days with marked improvement and the others need 2 cycles of IVIG with partial recovery.

Five patients presented by severe fatigue and progressive weakness of both lower and upper limbs with areflexia and hypoesthesia distally, they developed fever and cough 10 days after the neurological symptoms. EMG and NC were done and the electrophysiological findings of mixed demyelinating and axonal polyneuropathy picture were found. Those patients started treatment in form of 6 sessions of plasma exchange in three of them and the other two received 5 days of 1 gm methylpredinslone with marked improvement for all of them, when the respiratory symptoms manifested, laboratory tests and PCR done and confirmed COVID-19 diagnosis.

Four patients presented 30 to 40 days after their recovery form COVID-19 infection evidenced by 2 negative nasopharyngeal swaps with 48 h in between and discharged from hospital by gradual progressive weakness of both upper and lower limbs over 2 to 3 months duration mainly proximal muscles are affected more in lower limbs with areflexia. Two of them have bilateral lower motor neuron (LMN) facial and neck muscle weakness, and the other two patients had numbness in both lower limbs. EMG and NC were done and confirmed the diagnosis of chronic inflammatory demyelinating polyneuropathy **(**CIDP) for all of them fulfilling the electrophysiological criteria in form of markedly prolonged distal latencies (> 130% of ULN), markedly slowed conduction velocities (< 75% of the LLN), and markedly prolonged or absent late responses (> 130% of ULN), there is usually conduction block and/or temporal dispersion. The EMG examination showed MUP’s of reduced recruitment and no resting activities. All patients received 5 days of 1 gm methylpredinslone with marked improvement in one of them and minimal improvement in other three patients, with gradual withdrawal of steroids was planned to all of them.

With the start of 2nd wave of COVID 19 in Egypt, we added 19 patients to our study, 11 of them were in pediatric age group aged from 2 to 10 years old presented by picture of acute motor paralysis with ascending weakness affecting both lower and upper limbs with severe hypotonia and areflexia and 4 of them developed bilateral LMN facial. The condition preceded by gastrointestinal manifestations in 7 of them with vomiting, diarrhea with high grade fever. The other 4 had fever with cough preceding the weakness. EMG and NC done to all of them and revealed the typical picture of acute inflammatory demyelinating polyneuropathy **(**AIDP). All of them received IVIG with good response.

### Statistical analysis

The collected data was reviewed for completeness and logical consistency. Pre-coded data will be entered on the computer using Microsoft Office Excel software program for Windows 2019.Data was transferred to the Statistical Package for the Social Sciences **(**SPSS version 26**)** (IBM Corp., Armonk, NY, USA) to be statistically analyzed. Quantitative variables were described as mean, standard deviation, median, IQR. They were compared using Mann–Whitney U test, where p-value is significant if less than 0.05.Qualitative variables were described as frequency and percentage. They were compared using Chi-square test or Fisher's exact test accordingly where p-value is significant if less than 0.05. Exact test was used instead when the expected frequency is less than 5

## Results

Out of 42 patients with confirmed COVID-19, 23 (54.8%) in the first wave in the period from march to end of September 2020 and 19 (45.2%) patients in the second wave from October 2020 to end February 2021, 22 males (52.4%) and 20 females (48.6%), 28 adults (66.7%) their ages range from (35–55 years) and 14 in the pediatric group(33.3%), their ages range from (2–10 years). Clinical characteristics of studied patients illustrated in Table 1.

**Table 1:** Clinical characteristics of studied patients

		Frequency
Neuropathy	AIDP (motor and sensory)	25 (59.5%)
	AMSAN	8 (19%)
	Demyelinating with secondary axonal	5 (11.9%)
	CIDP like	4 (9.5%)
CT chest	Normal	2 (4.8%)
	Interstitial pneumonia	3 (7.1%)
	Interstitial pneumonia without parenchymal opacity	4 (9.5%)
	Bilateral GGO	33 (78.6%)
PCR	Positive	36 (85.7%)
CBC	Lymphopenia	30 (71.4%)
CRP	Elevated	31 (73.8%)
Outcome	Full Improvement	31 (73.8)
	No improvement and needed second treatment to improve	8 (19%)
	Minimal improvement	3 (7.1%)
	Total	42 (100%)

*AIDP* acute inflammatory demyelinating polyneuropathy, *AMSAN* acute motor sensory axonal neuropathy, *CIDP* chronic inflammatory demyelinating polyneuropathy, *CT* computed tomography, *GGO* ground glass opacity, *CBC* complete blood count, *CRP* C reactive protein

Regarding the comparison between the two waves of the pandemic, there is a significant difference between the 2 groups regarding the age with P value 0.00. Also, there is a significant difference regarding the mean age in the first wave comparing it with second wave, with P value 0.000, but there was no significant difference between both groups regarding the sex with P value 0.757 (Table 2).

**Table 2:** Comparisons between both waves regarding demographics

		Sex		P	Age group		P	Total
		Male	Female		Adults	pediatrics		
Wave	1st wave	13 (56%.5)	10 (43.5%)	0.757	20 (87%)	3 (13%)	0.003	23 100%
	2nd wave	9 (47.4%)	10 (52.6%)		8 (42.1%)	11 (57.9%)		19 100%

	Wave	n	Mean	SD	P
Age (in years)	1st wave	23	41.17	15.52	0.000
	2nd wave	19	19.63	18.43	

*n* number, *SD* standard deviation

There was no significant difference between both waves regarding the type of polyneuropathy (P value 0.193) and the outcome of the recovered patients (P value 0.084) (Table 3).

**Table 3:** Comparisons between both waves regarding the type of neuropathy and outcome

		Neuropathy type				P
		AIDP	AMSAN	Demyelinating with secondary axonal	CIDP like	
Wave	1st wave	11 (47.8%)	5 (21.7%)	3 (13.0%)	4 (17.4%)	0.193
	2nd wave	14 (73.7%)	3 (15.8%)	2 (10.5%)	0 (0.0%)	

		Outcome			P
		Full improvement	Minimal improvement	No improvement and needed second treatment to improve	
Wave	1st wave	14 (60.9%)	3 (13.0%)	6 (26.1%)	0.084
	2nd wave	17 (89.5%)	0 (0.0%)	2 (10.5%)	

*AIDP* acute inflammatory demyelinating polyneuropathy, *AMSAN* acute motor sensory axonal neuropathy, *CIDP* chronic inflammatory demyelinating polyneuropathy

Regarding the comparison between adults and pediatric groups, we found that the picture of neuropathy affecting the pediatric mainly is the AIDP rather than different types of neuropathy affecting the adults group with a significant difference between the two groups (P value 0.003) (Table 4), but there was no significant difference regarding the outcome of the neuropathy in treatment between the two groups (P value 0.129) (Table 5).

**Table 4:** Comparison between the adult and pediatric groups according to type of neuropathy

	Neuropathy type				Total	P
	AIDP	AMSAN	Demyelinating with secondary axonal	CIDP like		
Adult	11 (39.3%)	8 (28.6%)	5 (17.9%)	4 (14.3%)	28 (100%)	0.003
Pediatric	14 (100%)	0 (0%)	0 (0%)	0 (0.0%)	14 (100%)	

*AIDP* acute inflammatory demyelinating polyneuropathy, *AMSAN* acute motor sensory axonal neuropathy, *CIDP* chronic inflammatory demyelinating polyneuropathy

**Table 5:** Comparison between the adult and pediatric groups according to outcome of neuropathy

	Outcome			Total	P
	Full Improvement	Minimal improvement	No improvement and needed second treatment to improve		
Adult	18 (64.3%)	3 (10.7%)	7 (25%)	28 (100%)	0.129
Pediatric	13 (92.9%)	0 (0.0%)	1 (7.1%)	14 (100%)	

## Discussion

Coronaviruses are not primarily neurotropic viruses and their primary targets are respiratory and cardiovascular systems. The virus is attached to host cells through the Angiotensin-converting enzyme 2 (ACE-2) receptors leading to internalization and subsequent viral replication. This receptor is also found in glial cells in the Central Nervous System (CNS) and spinal neurons. The virus can invade peripheral nerves and lead to retrograde transfer via synapse mediated route to CNS [5, 6].

Also, the cytokine release syndrome (CRS), caused by an exacerbated recruitment and activation of macrophages, neutrophils, and natural killer cells (NK) in response to SARS-CoV-2 infection result in releasing many activated leukocytes during the inflammatory phase can lead to extensive tissue damage, including the peripheral nervous system, and appears to correlate with COVID-19 severity [7, 8].

COVID-19 may not directly invade nerves, its roots or the anterior horn cells as seen in polio virus or West Nile virus leading to their damage. Even the cerebrospinal fluid (CSF) and the polymerase chain reaction (PCR) for coronavirus in multiple reported cases of COVID-19 related GBS has been negative [9]. It is likely to be a post infectious or a para-infectious complication resulting from an aberrant immune response which can be considered a second mechanism explaining GBS in COVID-19 by production of antibodies against ganglioside components of the peripheral nerves, owing to molecular mimicry with surface antigens of the infectious pathogen [10].

Our study was conducted on 42 patients, included the first and second waves of the pandemic, through the period from March 2020 till the end of February 2021. We found that COVID and post COVID polyneuropathies with its different types and forms are of the common neurological complications of COVID virus infection that may affect and threaten the health if not taken in consideration.

In our study we reported that more than half of our patients (59.9%) presented with typical pattern of AIDP which was against Yaranagula and Koduri who reported that more than half of the patients in the their cohort had paraparesis [11]. The paraparetic variant is an uncommon form of GBS and constitutes about 5–10% of all GBS cases [12].

In the current study, most of our patients presented with demyelinating neuropathy rather than the axonopathy which was in accordance with Yaranagula and Koduri who found that the proportion of patients with demyelinating neuropathy was higher than previously reported COVID-19-associated neuropathies (84.62%) [11].

In a study done by Zhao and colleagues described two patients did not experience preceding fever, respiratory, or GI symptoms and GBS was the initial presentation [3], also Yaranagula and Koduri reported that three patients presented with acute neuropathy in the first week of their covid 19 infection suggesting a para-infectious process [11], as has been reported with Zika virus [13] and that agreed with our study.

In 2020, Virani and colleagues reported a 54-year-old male with confirmed COVID-19 and a history of fever, cough and *Clostridium difficile* colitis of recent onset developed difficulty breathing and weakness and diminished reflexes of arms and legs eventually diagnosed as GBS [10]. A 65 years old male was reported by Sedaghat and colleagues, with ascending quadrparesis, areflexia and bifacial palsy, after positive nasal swab and normal CSF, when EMG and nerve conductions were done on day 9 of weakness that revealed GBS variant-AMSAN [14].

El-Otmani and colleagues reported 70 years old female with quadriplegia, hypotonia and areflexia, that her MRI was normal but EMG and nerve conductions revealed axonal pattern of atypical GBS and diagnosed as ASAN, that it’s CSF protein was 100 mg/dL and normal WBCs [15] which agreed with our study.

Camdessanchi and colleagues reported a 64 years old male with flaccid paralysis and parasthesia. On day 5 EMG and NCV were done that revealed polyneuropathy of mixed demyelinating pattern, the CSF protein was 54 mg/dL [16].

In 2020, two other studies done by Scheidl and colleagues and Padroni and colleagues reported two patients with paraparesis and areflexia, EMG/NCS revealed demyelinating pattern and diagnosed as GBS and CSF proteins are elevated [17, 18].

Ottaviani and colleagues reported 66 years old Female with difficulty in walking, paraparesis and arefexia then progressed to quadriparesis with facial weakness. On day 10 EMG/NCS revealed mixed axonal and demyelinating patterns and diagnosed as GBS. The CSF protein was 108 mg/dL, normal WBCs and serum antiglycolipid antibodies were absent [19], which agreed with our study.

Abdelnour and colleagues reported 69 years old male presented with bilateral lower limb weakness and areflexia, brain and spine MRIs were normal, EMG/NCS were not performed however he was diagnosed as GBS, inspite that the CSF was not performed [20].

Caamaño DS and colleagues reported a 61 years old male with bilateral facial weakness, unresponsive blink reflex on both eyes. Brain MRI was normal. EMG/NCS were not performed. Diagnosed as atypical GBS variant with facial diplegia. The CSF protein was 44 mg/dL [21].

Toscano and colleagues reported 5 patients with GBS from three hospitals in northern Italy during the COVID-19 outbreak. In sum, GBS occurred 5 to 10 days after onset of COVID-19 symptoms, a typical interval. Clinical neurophysiology was consistent with axonal-type GBS in 3 cases and demyelinating-type in 2 patients. Post-Gadolinium MRI showed enhancement of caudal nerve roots in 2 patients and the facial nerve in one. All patients were treated with IVIG and Patients 1 and 3 received two cycles. A similar treatment was used in GBS patients during the MERS outbreak [22]^.^

With the emergence of more cases of polyneuropathies temporally linked to SARS-CoV-2 infection, we can understand the improvement of such patients on steroids, plasma exchange and IVIG. We should gain a better understanding of the underlying pathophysiology and potential therapeutic options of GBS related to COVID-19.

Several therapeutic options are still under study, with the intent to stabilize the immune system in COVID-19 and either prevent or minimize the consequences of this storm, as reviewed by Diamanti and colleagues [23].

Since these neuropathies are treatable and they pose increased morbidity and mortality, physicians working with COVID-19 patients must be aware of this association.

In our study, we noticed different types of polyneuropathy which vary according to age groups and also it may be the initial COVID symptoms between Group A and Group B, this could be due to the genetic change in the corona virus phenotype or change in the autoimmune response of the body to the virus in the second wave.

The presentation of the neurological complications attacked the younger age in the second wave may be due to the eased of the global restrictions of closure, opening of the schools and nurseries, making the children in this age group more vulnerable for infection, or may the adults gained immunity from previous exposure to the first coronavirus season as they were more exposed for infection for the sake of keeping their works and the individual financial income.

## Conclusions

We should gain a better understanding of the underlying pathophysiology and therapeutic options of polyneuropathies related to COVID-19, which will have an impact on the treatment of the COVID related respiratory failure presenting with neuropathy.

## Data Availability

The datasets generated and/or analyzed during the current study are not publicly available due to the current Cairo University regulations and Egyptian legislation but are available from the corresponding author on reasonable request and after institutional approval.

## Abbreviations

COVID-19: Coronavirus disease 2019
SARSCoV-2: Severe acute respiratory syndrome coronavirus 2
DM: Diabetes mellitus
HCV: Hepatitis C virus
EMG: Electromyography
NC: Nerve conduction
CT: Computed tomography
CBC: Complete blood count
DL: Distal latency
ESR: Erythrocyte sedimentation rate
CRP: C reactive protein
ULN: Upper limit of normal
LLN: Lower limit of normal
CMAP: Compound motor action potentials
SNAPs: Sensory nerve action potentials
MRI: Magnetic resonance imaging
PCR: Polymerase chain reaction
IVIG: Intravenous immunoglobulin
MUP’s: Motor unit potentials
CIDP: Chronic inflammatory demyelinating polyneuropathy
AIDP: Acute inflammatory demyelinating polyneuropathy
AMSAN: Acute motor sensory axonal neuropathy
MERS: Middle East Respiratory Syndrome
ACE-2: Angiotensin-converting enzyme 2
CNS: Central nervous system
GBS: Gullian Barre Syndrome
GGO: Ground glass opacity
IVIG: Intravenous immunoglobulins
